# Comment on “World's First Clinical Case of Gene-Activated Bone Substitute Application”

**DOI:** 10.1155/2017/4383926

**Published:** 2017-07-12

**Authors:** Albert A. Rizvanov, Ruslan F. Masgutov

**Affiliations:** ^1^Kazan (Volga Region) Federal University, Kazan, Russia; ^2^Republic Clinical Hospital, Kazan, Russia

We read with great interest the paper entitled “World's First Clinical Case of Gene-Activated Bone Substitute Application” in Case Reports in Dentistry [1]. We would like to draw your attention to our recent study “Use of Gene-Activated Demineralized Bone Allograft in the Therapy of Ulnar Pseudarthrosis. Case Report” in BioNanoScience [2]. Despite the different pathologies in these two papers, it is interesting to compare them.

Firstly, it is important to understand that there is currently only one approved gene plasmid-based therapy on the world market. Indeed several authors in the Deev et al. paper actively participated in the development [3] of the VEGF165 encoding plasmid for the stimulation angiogenesis in ischemic limbs. Since angiogenesis is very important for osteogenesis, the authors naturally decided to use this plasmid. Secondly, the authors demonstrate the successful widening of indications for an approved treatment for osteogenesis. However, this line of research is limited in its choice of therapeutic growth factors.

This is why we focused on a completely new approach for gene activation of a demineralized bone allograft. We created a dual cassette expression plasmid, encoding for both proangiogenic vascular endothelial growth factor 165 (VEGF165) and proosteogenic bone morphogenetic protein 2 (BMP2). We believe that expression of both these growth factors not only stimulated angiogenesis, but also enhanced osteogenesis at the site of the bone graft transplantation.

Similar to Deev et al., we demonstrated the effectiveness of gene-activated bone transplants in stimulating osteogenesis which, in our study, resulted in callus formation at the site of ulnar pseudarthrosis. Besides the many in vitro and in vivo animal studies expertly reviewed by Deev et al. [4], to our knowledge these two clinical case reports are the first demonstrating the safety and efficacy of transplanting gene-activated bone grafts for the treatment of various bone pathologies, paving the way for additional clinical trials. Comparing proosteogenic effects of single or combinations of therapeutic growth factors and the mode of application of plasmid DNA on various natural, artificial, and demineralized bone grafts, with different types of bone defects, would ultimately identify more optimal therapeutic strategies.
